# Analysis of Rhizosphere Microbial Diversity Among Different Maize Varieties in the Hexi Corridor

**DOI:** 10.3390/microorganisms14081746

**Published:** 2026-08-08

**Authors:** Dong-Ping Li, Feng Zhang, Pratiksha Singh, Bin Yang, Ai Ge, Rajesh Kumar Singh, Dao-Jun Guo

**Affiliations:** 1 Key Laboratory of Hexi Corridor Resources Utilization of Gansu, Hexi University, Zhangye 734000, China; lidongping0201@126.com (D.-P.L.);; 2 College of Life Sciences and Engineering, Hexi University, Zhangye 734000, China; 3 Key Laboratory of Sugarcane Biotechnology and Genetic Improvement, Guangxi Academy of Agricultural Sciences, Nanning 530007, China; 4 School of Agriculture Science, LNCT University, Bhopal 462042, Madhya Pradesh, India; 5 College of Agriculture and Ecological Engineering, Hexi University, Zhangye 734000, China

**Keywords:** maize, rhizosphere, diversity, genotype, microbial community structure

## Abstract

Rhizosphere microorganisms have a significant impact on plant growth and development. To clarify the regulatory mechanisms underlying maize genotype effects on rhizosphere soil physicochemical factors and microbial communities in the Hexi Corridor of China, this study used seven maize varieties (Wugu 305, Xianyu 335, Zhengdan 958, Jingke 968, Yufeng 303, Dedan 1104, and Qinfeng 876) as experimental materials. Rhizosphere soil physico-chemical indicators were measured, and high-throughput sequencing was used to analyze the diversity, structure, unique bacterial communities, and metabolic functions of rhizosphere fungi and bacteria. Correlation analysis was also conducted using soil environmental factors. The results showed that the pH of the tested maize rhizosphere soil was weakly alkaline (7.96–8.10); significant varietal differences were observed in total nitrogen, available nitrogen, and available phosphorus, while no significant differences were observed in organic matter and total phosphorus among maize varieties. Dedan 1104 and Jingke 968 had the highest fungal richness, diversity, and evenness at the phylum and genus level, according to an alpha-diversity study of maize rhizosphere soil microorganisms, which revealed that variation did not affect fungal community richness. Zhengdan 958 has the highest microbial richness at the bacterial phylum level. At the bacterial genus level, Dedan 1104 had the highest microbial abundance. Ascomycota is the predominant fungal phylum, with Proteobacteria, Acidobacteria, and Chloroflexi being the core bacterial phyla. Venn analysis confirmed strong conservation of fungal community composition across various maize varieties; abundant bacterial-specific taxa and greater genotype-driven differentiation. Furthermore, significant differences were found in the enriched microbial communities in the rhizosphere among different maize genotypes, with fungal metabolic functions strongly influenced by maize genotype, and the distribution of core bacterial functional enzymes tending towards homogeneity. Available phosphorus and alkaline-available nitrogen were the core environmental factors regulating the structure of rhizosphere fungal and bacterial communities, and the dominant functional microorganisms were significantly positively correlated with these nutrients. This study provides a theoretical basis for screening high-quality seed maize varieties and regulating rhizosphere soil microecology.

## 1. Introduction

The Hexi Corridor, a narrow region of arid to semi-arid land in northwestern China, serves as China’s primary base for maize seed production, supplying more than half of China’s hybrid maize seeds from approximately 100,000 hectares of cultivated land [1,2]. Due to abundant sunlight, significant diurnal temperature variation, and a temperate desert climate that restricts the spread of seed-borne pathogens, this region serves as a vital center for commercial seed production in China [3,4]. However, there is growing uncertainty about the ecological viability of this extreme monoculture system. Long-term monoculture of seed maize, which can persist for more than 30 years in some areas, has resulted in significant soil deterioration, disrupted nutrient cycling, and increased incidence of soil-borne disease [5,6,7]. The primary ecological interface that influences the health of farming systems is the rhizosphere. Plant root exudates can select, filter, and organize complex microbial communities composed of bacteria and fungi, thanks to this dynamic soil compartment [8,9,10]. The rhizosphere-associated component of the plant microbiome, in particular, is essential for controlling nutrient uptake, boosting stress tolerance, and inhibiting pathogens [11,12]. Both stochastic environmental filtration and deterministic host-mediated selection work together to control the formation of rhizosphere bacterial and fungal populations [13]. Numerous endogenous plant variables, such as the makeup of root exudates, genotype-specific chemical signaling pathways, and various stages of maize plant development, govern host-dependent deterministic processes. The other major driver, stochastic environmental filtering, is shaped by extrinsic abiotic factors such as soil moisture content, edaphic characteristics, and regional climatic aridity gradients [14,15]. Ecological filters in arid ecosystems function with heightened intensity. Drought stress, increased salinity, and extreme temperature regimes impose significant physiological constraints on plant hosts and their associated microbiomes, resulting in predictable changes in community composition and taxonomic diversity [16,17,18]. According to recent research, rhizosphere bacterial communities are systematically altered by increasing dryness, and community β-diversity exhibits substantial relationships with climate gradients [19]. Additionally, the Hexi Corridor’s desert soils exhibit unique biogeographical patterns in the diversity of bacterial and eukaryotic microorganisms, which are mainly determined by soil physicochemical characteristics and spatial autocorrelation [20,21,22]. However, most existing research has focused on natural or uncropped ecosystems, leaving a significant knowledge gap regarding the rhizosphere microbiome’s response to host genotype variation within agricultural systems in this arid region.

Recent studies on maize rhizosphere microbial ecology within seed production systems have primarily focused on temporal comparisons, specifically investigating microbial community changes across continuous cropping chronosequences [23,24]. An increasing enrichment of the pathogenic genus *Fusarium* and a depletion of beneficial taxa like *Mortierella* and *Chrysosporium* have been seen in fungal and bacterial communities throughout successive years of mono-maize farming [25]. In the Hexi Corridor, follow-up studies have validated similar nonlinear succession patterns when cropping duration exceeds two decades [26,27]. The impact of host genetic identity, including different root exudation profiles, architectural features, and physiological signatures among commercial maize varieties, on rhizosphere microbial community structure is still mostly unknown, despite advances in our understanding of temporal dynamics.

This study aims to conduct a comprehensive analysis based on amplicon sequencing of the rhizosphere bacterial (16S rRNA) and fungal (ITS) community composition and diversity of several commercially cultivated maize varieties in the Hexi Corridor. Specifically, its objectives are: (i) characterize and compare diversity patterns of bacterial and fungal communities among different maize varieties under identical environmental and management conditions; (ii) identify taxonomic signatures at both phylum and genus levels that differentiate variety-specific rhizosphere assemblages from one another and from bulk soil reference communities; (iii) predict functional profiles of the differentially abundant taxa to evaluate whether variety-specific microbiomes harbor distinct ecological potentials related to nutrient cycling and plant health promotion.

## 2. Materials and Methods

### 2.1. Collection of Rhizosphere Soil from Seed Maize Varieties

Rhizosphere soil samples were collected from seven seed-production maize varieties at the National Maize Seed Production Demonstration Base in Linze County, Zhangye City, Gansu Province, China. The specific geographic coordinates of the maize samples are presented in Supplementary Table S1. The site is located at an average altitude of approximately 1400 m, with an annual effective accumulated temperature (≥10 °C) of 3053 °C and a frost-free period of 160 days. The region experiences an arid climate, low precipitation, an annual average rainfall of 120 mm, an annual average temperature of 8.1 °C, and a daily average temperature of 14.9 °C. Additionally, the area exhibits a large diurnal temperature range and a long duration of sunshine. At the sampling site, all maize hybrids were sown in late April at a density of 75,000 plants per hectare, with rows spaced 40 cm apart and plants spaced 33 cm apart. Agronomic management for all plots followed local recommended standards, utilizing integrated drip irrigation and fertigation with split applications of 240 kg·ha^−1^ nitrogen, 100 kg·ha^−1^ phosphorus pentoxide, and 60 kg·ha^−1^ potassium oxide, in addition to integrated pest and disease management. Soil samples were collected during the peak growth stage of maize. At each sampling time point, healthy maize plants of uniform vigor were randomly selected from each plot and excavated to a soil depth of 30 cm using a sterilized stainless steel spade to minimize cross-contamination between plots. Soil adhering to the maize root systems was removed using a sterile brush, and the rhizosphere soil was transferred into sterile bags and stored at −80 °C for subsequent analysis. Three replicate rhizosphere soil samples were collected for each maize variety: WuGu 305 (WG305: WG305_1, WG305_2, WG305_3), XianYu 335 (XY335: XY335_1, XY335_2, XY335_3), ZhengDan 958 (ZD958: ZD958_1, ZD958_2, ZD958_3), JingKe 968 (JK968: JK968_1, JK968_2, JK968_3), YuFeng 303 (YF303: YF303_1, YF303_2, YF303_3), DeDan 1104 (DD1104: DD1104_1, DD1104_2, DD1104_3), and QinFeng 876 (QF876: QF876_1, QF876_2, QF876_3).

### 2.2. Testing of Soil Physicochemical Properties

Using established analytical techniques, the physicochemical characteristics of the maize rhizosphere soil were ascertained by weighing around 300 g of each soil sample. A calibrated digital pH meter (Mettler Toledo FE28, Zurich, Switzerland) was used to measure soil pH in a soil-water suspension at a 1:2.5 (*w*/*v*) ratio [28]. The alkali-hydrolysis diffusion method was used to assess accessible nitrogen (AN), and the semi-micro Kjeldahl method was used to determine total nitrogen (TN) [29]. The alkali fusion-molybdenum antimony-ascorbic acid colorimetric method was used to calculate total phosphorus (TP) [30]. Using a 0.5 M NaHCO_3_ solution (Olsen technique, pH 8.5), available phosphorus (AP) was extracted and colorimetrically quantified at 700 nm [31]. The potassium dichromate volumetric method (external heating) was used to measure soil organic matter [32]. Every measurement was carried out three times.

### 2.3. Total DNA Extraction and Quality Assessment

Aliquots of 0.5 g soil were weighed, and total microbial DNA was extracted using the TIANamp Soil DNA Kit (DP336, Tiangen Biotech Co., Ltd., Beijing, China). Samples were homogenized in Lysis Matrix E tubes (Tiangen Biotech Co., Ltd., Beijing, China) containing ceramic and silica beads with CLS-VF buffer, followed by bead-beating at 6.0 m·s^−1^ for 40 s using a FastPrep-24 instrument (MP Biomedicals, Santa Ana, CA, USA). DNA was purified using silica-based spin columns and eluted in buffer TE. DNA quality was assessed by 1.5% (*w*/*v*) agarose gel electrophoresis, and concentration was determined with a NanoDrop 2000 spectrophotometer (Thermo Fisher Scientific, Waltham, MA, USA). Purity was verified by A260/A280 (1.8–2.0) and A_260_/A_230_ (>1.7) ratios. Extracted DNA samples were stored at −80 °C for subsequent analysis.

### 2.4. PCR Amplification of Bacterial 16S rRNA and Fungal ITS Regions

For bacterial community profiling, the V3–V4 hypervariable region of the 16S rRNA gene was amplified using the universal primer pair 341F (5′-CCTACGGGNGGCWGCAG-3′) and 806R (5′-GGACTACHVGGGTWTCTAAT-3′) [33]. For fungal community profiling, the internal transcribed spacer (ITS) region was amplified using primers ITS1F (5′-CTTGGTCATTTAGAGGAAGTAA-3′) and ITS4R (5′-TCCTCCGCTTATTGATATGC-3′) [34]. Each PCR reaction (30 μL total volume) consisted of 12.5 μL of 2× KAPA HiFi HotStart ReadyMix (KAPA Biosystems, Wilmington, MA, USA), 0.5 μL of each forward and reverse primer (10 μM), 2.0 μL of template DNA (~10–15 ng), 1.5 μL of BSA solution (0.1%), and nuclease-free water to the final volume. PCR was performed using sterile water as the negative control. The same conditions and chemicals were used for the amplification of every experimental sample. The lack of bands in the gel image from the negative control PCR amplification ensured that the experimental data were accurate and prevented false positives by confirming that the PCR reagents were not contaminated.

### 2.5. Library Preparation and High-Throughput Sequencing

Triplicate PCR amplicons from each sample were pooled and purified using AMPure XP magnetic beads (Beckman Coulter, Brea, CA, USA) to remove primer dimers and non-specific products. Purified amplicons were quantified using a Qubit 3.0 fluorometer and the Qubit dsDNA BR Assay Kit (Invitrogen, Life Technologies, Carlsbad, CA, USA). Indexed libraries were combined in equimolar amounts according to amplicon concentration, and the pooled library was sequenced in paired-end mode (2 × 300 bp) on an Illumina NovaSeq platform (Illumina, Inc., San Diego, CA, USA) at Majorbio Bio-pharm Technology Co., Ltd., Shanghai, China. Operational Taxonomic Units (OTUs) with fewer than 20 sequences per group or with a count of 5 or fewer in at least three samples were excluded to improve the reliability and accuracy of OTU detection.

### 2.6. Bioinformatics Analysis and Microbial Diversity Assessment

Taxonomic classification, sequence denoising, and quality control were carried out using the Quantitative Insights Into Microbial Ecology 2 (QIIME 2) platform (v2023.7) [35]. Demultiplexing and quality filtering of raw paired-end reads were made easier by the DADA2 plugin. Reads that contained ambiguous bases (‘N’) or were less than the specified truncation length were eliminated. At locations where the median quality score fell below Q20, forward and reverse reads were cut. Amplicon sequence variants (ASVs) were inferred using the DADA2 internal error model, which allows single-nucleotide resolution without arbitrary clustering limits. Chimeric sequences were identified and removed using the DADA2 consensus-based method [36]. A pre-trained naive Bayes classifier was used to taxonomically classify representative ASV sequences. The SILVA reference database (v138.1) was used to classify bacterial 16S rRNA sequences using a 99% identity criterion. The taxonomic assignment of fungal ITS sequences was performed at the species level using the UNITE dynamic database (v9.0). Downstream analyses did not include ASVs categorized as mitochondria, chloroplasts, or unassigned at the kingdom level.

Rarefaction curves were created to assess the adequacy of the sequencing depth. Five indices were used to evaluate alpha diversity (within-sample diversity), including the Simpson index (dominance) and the Shannon index (diversity integrating richness and evenness). To guarantee comparability, rarefied feature tables were created at a consistent sequencing depth equal to the lowest read count across all retained samples. One-way analysis of variance (ANOVA) followed by Tukey’s honestly significant difference (HSD) test or the non-parametric Kruskal–Wallis test when normality (Shapiro–Wilk test) or homogeneity of variance (Levene’s test) assumptions were broken, was used to test differences in alpha diversity.

Both phylogenetic (unweighted and weighted UniFrac distance) and non-phylogenetic (Bray–Curtis dissimilarity) metrics were used to compute beta diversity (between-sample community dissimilarity). Permutational multivariate analysis of variance (PERMANOVA) with 999 permutations was used in the vegan R package (v2.6-4) to evaluate the statistical significance of group separation [37]. To ensure that observed differences were not due to unequal within-group variability, multivariate homogeneity of group dispersions was assessed using the betadisper function prior to PERMANOVA.

### 2.7. Data Analysis

One-way analysis of variance (ANOVA) was conducted on soil bacterial and fungal alpha diversity indices and soil physicochemical characteristics using SPSS 24.0. Analysis of microbiome composition with bias correction (ANCOM-BC) was used to identify species that were differentially abundant among sample groups [38]. Additionally, using default parameters (LDA score ≥ 3.5, *p* < 0.05), biomarker taxa distinguishing particular growth stages were identified using linear discriminant analysis effect size (LEfSe) [39]. The relationships between dominant microbial taxa (relative abundance > 1%) and measured soil physicochemical characteristics were assessed using Spearman’s rank correlation [40]. R software (v4.3.1) and RStudio (v2023.06.1) were used for all statistical analyses and data visualizations. Unless otherwise noted, statistical significance was determined as *p* < 0.05. The ggplot2 (v3.4.2) and pheatmap (v1.0.12) software were used to create the figures. Data preprocessing and summarization were performed with Microsoft Excel. Unique operational taxonomic unit (OTU) analyses of microbial communities were conducted using R 4.3.1 with the Vegan, tidyverse, and ggplot2 packages. Fungal trophic modes were identified using FunGuild [41], and soil bacterial functions were examined through KEGG pathway analysis with Tax4Fun [42].

## 3. Results

### 3.1. Physicochemical Properties of Maize Rhizosphere Soil

The rhizosphere soil pH of the seven seed maize varieties ranged from 7.96 to 8.10, suggesting a generally slightly alkaline state (Table 1). Total nitrogen (TN), accessible nitrogen (AN), and accessible phosphorus (AP) showed significant varietal differences *(p* < 0.05), whereas total phosphorus (TP) and soil organic matter (OM) showed no significant variations (*p* > 0.05) among the maize cultivars. Compared to the other kinds, WG305 and DD1104 have the highest rhizosphere TN concentration (Table 1). In terms of AN content, XY335 recorded the lowest value, whereas ZD958 and DD1104 had noticeably larger levels than XY335 and QF876. YF303 and WG305 had the highest levels of AP, whilst XY335 had the lowest (Table 1). WG305 had the highest organic matter content and XY335 the lowest, but the differences between the varieties were not statistically significant. Similarly, QF876 had the highest mean value and XY335 the lowest, despite no significant differences in TP (Table 1).

### 3.2. Alpha Diversity of Rhizosphere Soil Microorganisms in Different Maize Varieties

The phylum-level Sobs and Bootstrap indices of fungal communities in the rhizosphere soils of several maize varieties did not differ significantly (Table 2). On the other hand, ZD958 had the lowest Shannon index, and DD1104 had the highest. ZD958 and WG305 had lower community evenness than DD1104, XY335, and JK968, as measured by the Simpson index. While maize variety had no effect on fungal community richness at the phylum level, there were notable variations in community evenness and diversity among varieties, with DD1104 showing the highest diversity (Table 2). At the fungal genus level, microbial richness indices showed considerable varietal differentiation: XY335, WG305, and ZD958 had the lowest richness, while JK968 and DD1104 had much higher Sobs and Bootstrap indices than the other five varieties (Table 3). Compared with WG305, XY335, ZD958, and QF876, the Shannon index was much higher for YF303, DD1104, and JK968, and significantly lower for XY335 and ZD958; the Simpson index was lower for JK968, YF303, and DD1104 (Table 3). In conclusion, compared with other maize cultivars, the rhizosphere soil fungal communities of JK968 and DD1104 showed notably higher genus-level richness, diversity, and evenness.

ZD958 had the highest microbial richness (Sobs and Bootstrap indices) for rhizosphere bacteria at the phylum level (*p* < 0.05) among the seven maize types, whereas YF303, WG305, and JK968 had significantly lower richness (Table 4). In terms of community diversity, XY335 had a much higher Shannon index than the other kinds, whereas YF303 and JK968 had the lowest diversity. At the phylum level, YF303 had the highest Simpson index, suggesting the worst community evenness. In general, XY335 showed the highest phylum-level community diversity and evenness, while ZD958 contained the greatest number of bacterial phyla (Table 4). In general, the genus-level bacterial microbial richness indices were significantly greater than the phylum-level ones. While XY335 had the lowest richness indices, DD1104 had much higher Sobs and Bootstrap indices than the other types (Table 5). At the bacterial genus level, JK968 showed the highest Shannon diversity index, followed by QF876 and XY335, which had the lowest. Simpson indices did not change significantly between groups, suggesting similar community evenness (Table 5). In conclusion, DD1104 had the highest bacterial abundance at the bacterial genus level.

### 3.3. Rhizosphere Soil Microbial Community Structure of Different Maize Varieties

**Figure 1:**
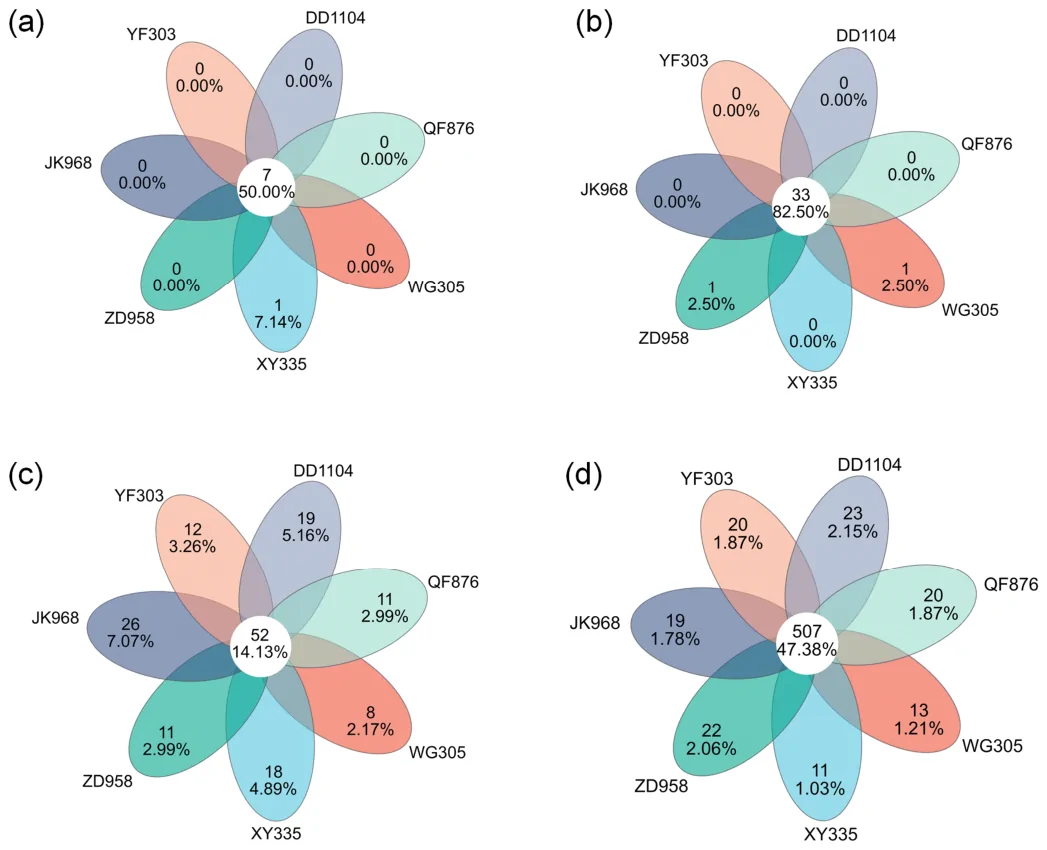
Venn diagram analysis of rhizosphere microbial communities of different maize varieties. (**a**) Rhizosphere soil fungal communities of different maize varieties at the phylum level. (**b**) Rhizosphere soil bacterial communities of different maize varieties at the phylum level. (**c**) Rhizosphere soil fungal communities of different maize varieties at the genus level. (**d**) Rhizosphere soil bacterial communities of different maize varieties at the genus level.

The rhizosphere fungal communities of the seven maize varieties showed low proportions of variety-specific taxa and relatively high proportions of common taxa. Seven common fungal phyla were found at the phylum level, making up 50.00% of all fungal phyla; only XY335 had one endemic phylum, whilst the other types had none (Figure 1a). In total, 33 common bacterial phyla were found, accounting for 82.50% of the total bacterial phyla. Only ZD958 and WG305 each had one endemic bacterial phylum; no endemic bacterial phylum was found in the other varieties, indicating strong conservation of rhizosphere bacterial composition among various maize genotypes (Figure 1b). At the genus level, Specific fungal taxa were present in each variety, with genotype-driven community differences being more noticeable. Only 52 common fungal genera, or 14.13% of all fungal genera, were found at the genus level; all maize varieties had endemic fungal genera, ranging from 8 to 26, with JK968 having the most and WG305 having the fewest (Figure 1c). There were 507 bacterial genera at the genus level, making up 47.38% of all bacterial genera. There were 11–23 species-specific bacterial genera, with DD1104 having the most and XY335 having the fewest. Compared to the phylum level, the proportion of common components among bacterial genera was dramatically enhanced (Figure 1d).

**Figure 2:**
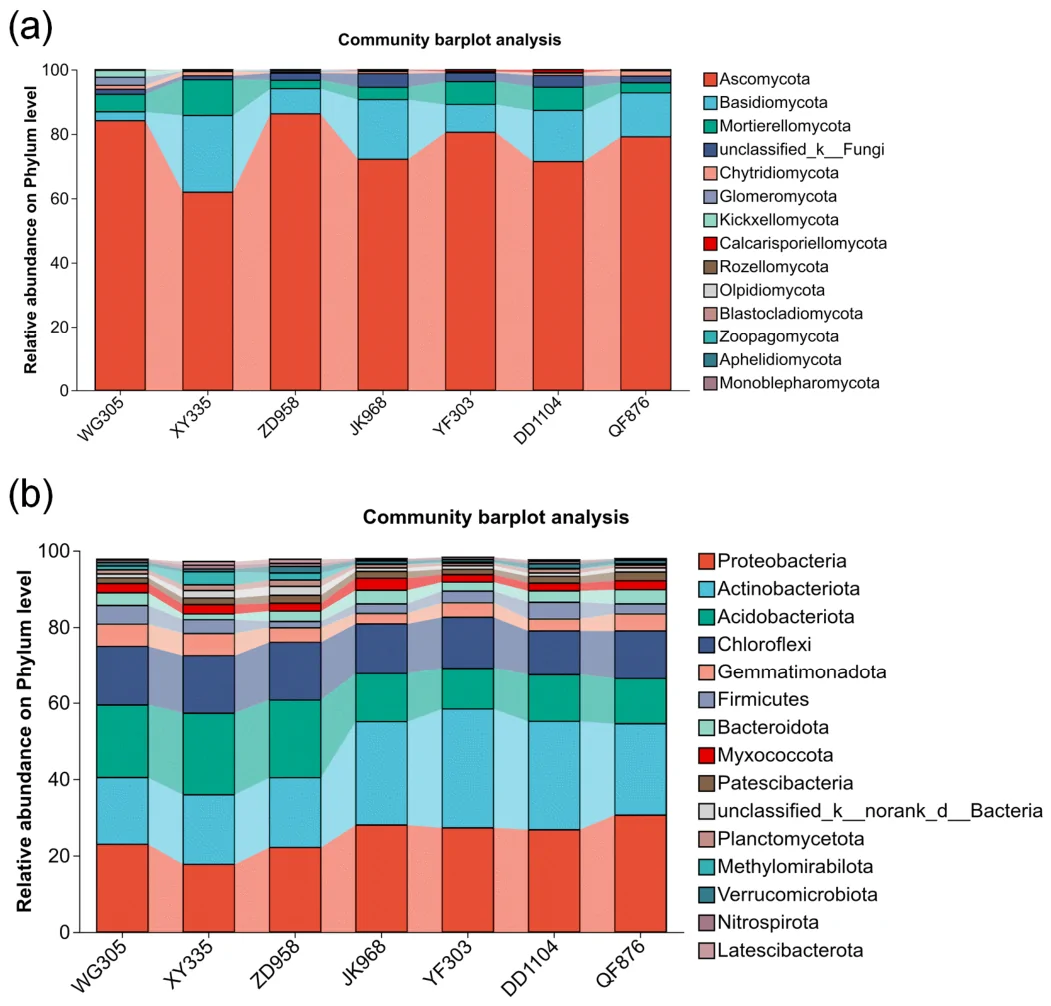
Rhizosphere soil microbial community structure of different maize varieties at the phylum level. (**a**) Fungal community structure. (**b**) Bacterial community structure.

Phylum-level fungal community structure analysis showed that Ascomycota was the absolutely dominant fungal phylum in the rhizosphere soil of all maize varieties, with a relative abundance of over 60% in each sample; Basidiomycota and Mortierellomycota were the second most dominant groups, while the remaining 12 low-abundance fungal phyla and unclassified fungal groups accounted for a negligible proportion (Figure 2a). Several phyla dominated the bacterial community in the maize rhizosphere, with Proteobacteria, Actinobacteriota, Acidobacteriota, and Chloroflexi accounting for more than 70% of the total relative abundance. *Bacillus*, Firmicutes, and Bacteroidota were moderately abundant, while the abundance of other phyla, such as Nitrospirota and Latescibacterota, was typically low (Figure 2b).

**Figure 3:**
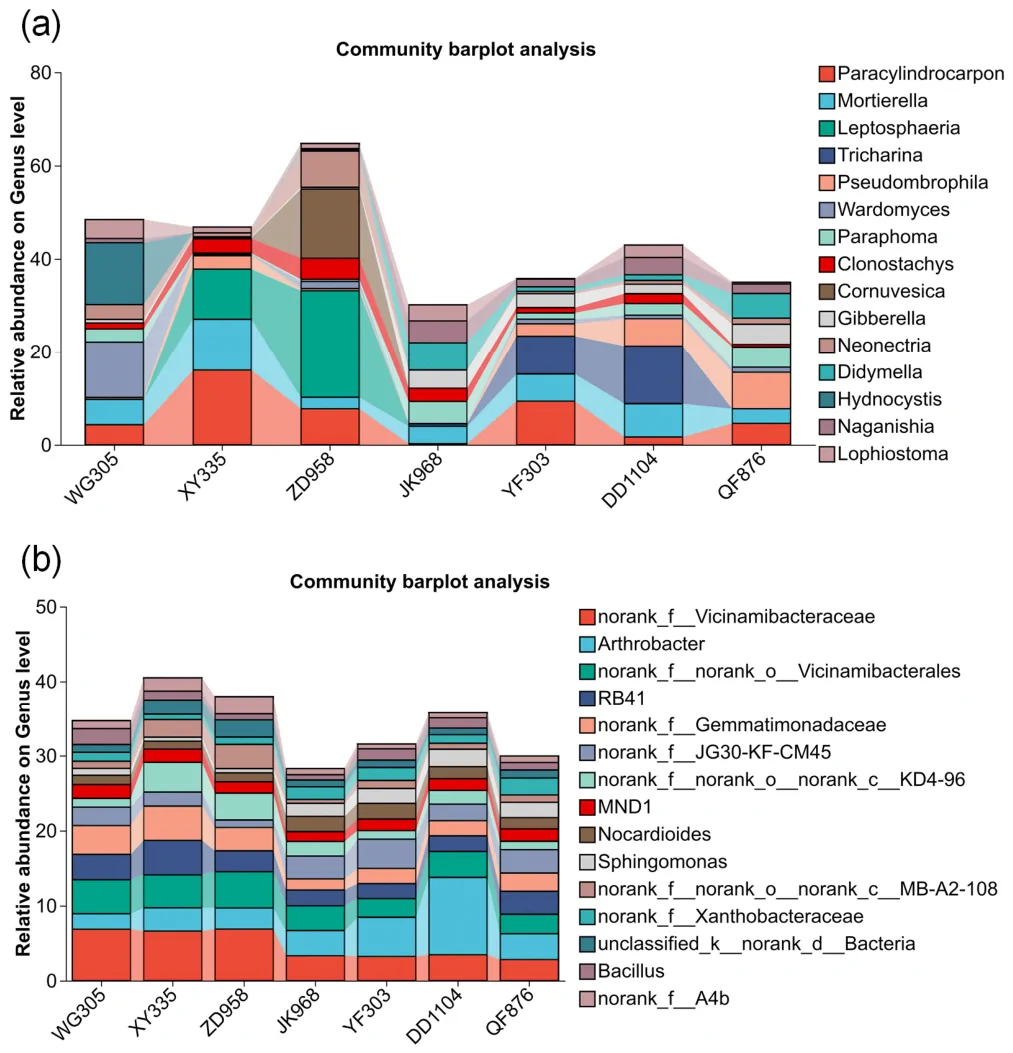
Rhizosphere soil microbial community structure of different maize varieties at the genus level. (**a**) Fungal community structure. (**b**) Bacterial community structure.

Based on a genus-level analysis of the first 15 fungal communities, the most common genera across all maize rhizosphere microorganisms were *Paracylindrocarpon*, *Mortierella,* and *Leptosphaeria*; the other genera were linked to low to medium abundance. Fungal genera abundance differentiation was significantly mediated by maize genotype: *Paracylindrocarpon*, *Leptosphaeria*, *Cornuvesica*, and *Naganishia* were particularly enriched in the rhizosphere of ZD958, with a total abundance of these dominant genera significantly higher than in other varieties; JK968 had a generally low abundance of all fungal genera, with a more even distribution of community components; XY335, DD1104, WG305, and QF876 had moderate abundance of dominant fungal genera, and the community structure demonstrated significant differentiation (Figure 3a). The top 15 rhizosphere bacterial genera included a large number of norank groups with undetermined taxonomic status and typical rhizosphere functional genera, with the core dominant groups being norank_f_Vicinamibacteraceae, *Arthrobacter*, and RB41. The enrichment patterns of rhizosphere bacterial genera differed significantly among different maize genotypes: XY335 had the highest total abundance of dominant bacterial genera, with *Bacillus* being significantly enriched; in DD1104, the relative abundance of Arthrobacter was much higher than that of other varieties; in JK968, the abundance of various bacterial genera was generally low; *Bacillus* was present in the rhizosphere soil of all maize varieties (Figure 3b).

### 3.4. Differences in Rhizosphere Soil Microorganisms Among Different Maize Varieties

**Figure 4:**
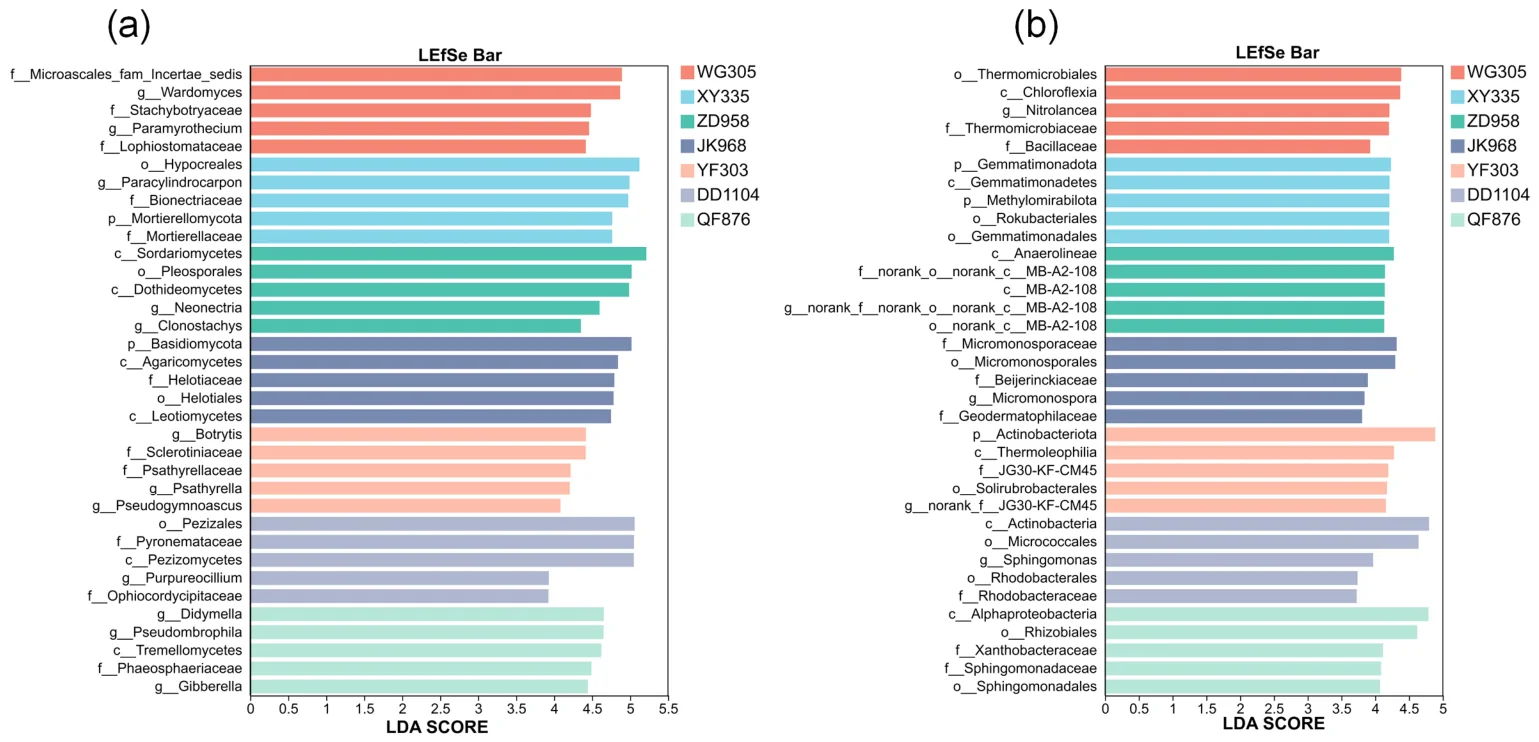
LDA discrimination of LEfSe analysis of differential microorganisms in rhizosphere soil of different maize varieties (LDA score ≥ 3.5, *p* < 0.05). (**a**) LDA discrimination of different fungi; (**b**) LDA discrimination of different bacteria.

Differentially enriched rhizosphere fungal microorganisms across maize varieties were screened at the phylum and genus levels using LEfSe analysis (LDA > 3.5). The enhanced, differentially enriched microbial communities of various maize genotypes showed distinct differentiation (Figure 4a). Lower-order fungal groups like *Wardomyces*, Microascales, and Paramyrothecium were specifically enriched by WG305; Mortierellomycota and *Paracylindrocarpon* were significantly enriched by XY335; Sordariomycetes, Pleosporales, and Dothideomycetes were enriched by ZD958, which had the highest LDA score and strongest indicative effect; related groups of Basidiomycota and Agaricomycetes were enriched by JK968, respectively. The unique indicator fungus present in each variety across taxonomic levels demonstrated the remarkable specificity of maize genotypes for rhizosphere fungal communities (Figure 4a). LEfSe bacterial analysis revealed differential bacterial communities among the maize varieties. WG305 showed core indicator bacteria in the phylum Chlorella, order Thermomycetes, and Nitrolancea; XY335 significantly enriched in the phylum Gemmatimonadota and Methylomirabilota; ZD958 enriched in the class Anaerolineae and several unclassified norank bacterial groups; JK968 specifically enriched in the family Micromonosporaceae and Micromonospora; YF303 enriched in the class Thermomycetes of the phylum Actinobacteriota; DD1104 and QF876 showed characteristic differential bacterial communities in Sphingomonas and Sphingomonasaceae of the class Alphaproteobacteria, respectively (Figure 4b).

### 3.5. KEGG Function of Rhizosphere Soil Microorganisms in Different Maize Varieties

**Figure 5:**
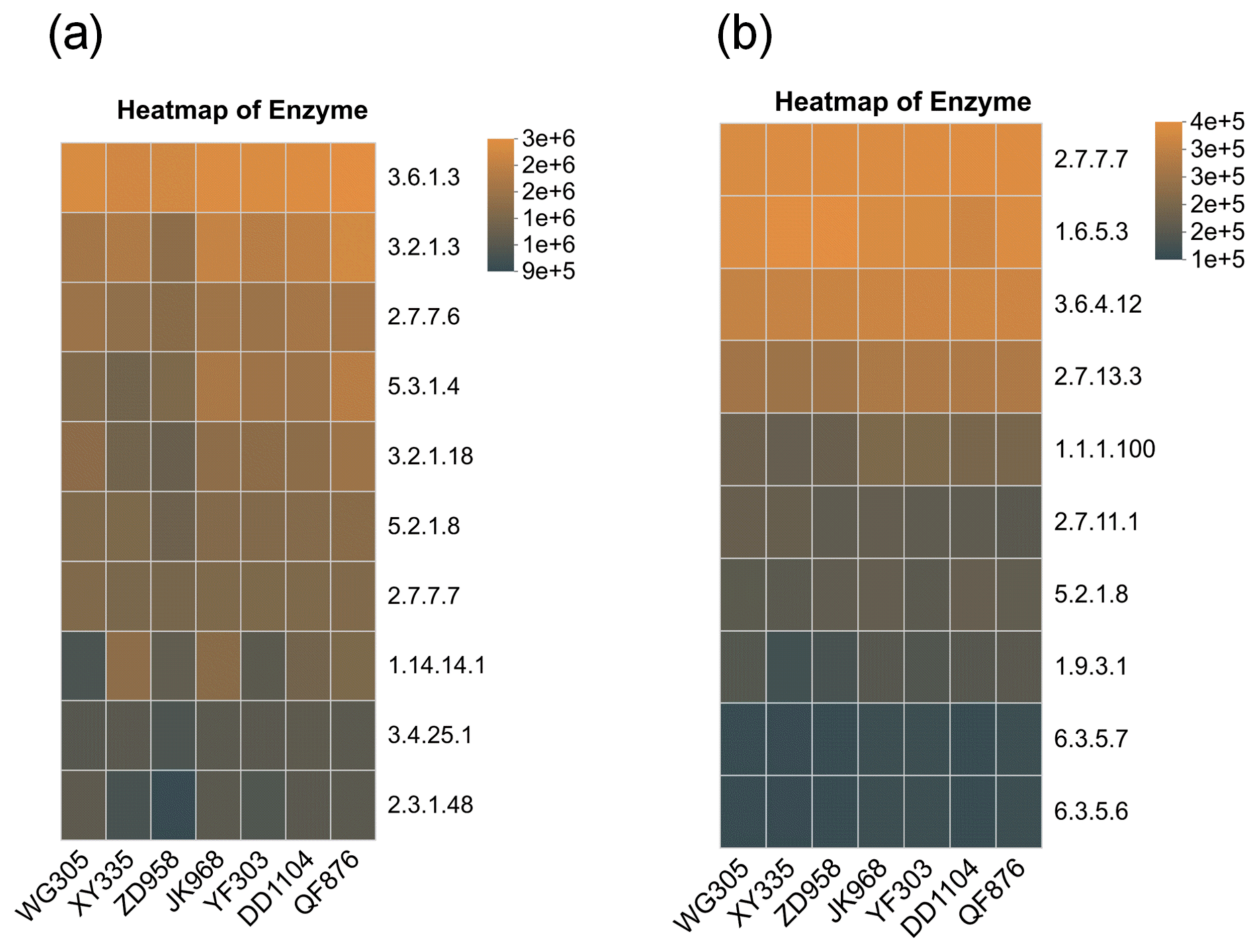
KEGG function of maize rhizosphere soil microorganisms. (**a**) Function of KEGG fungi in maize rhizosphere soil. (**b**) Function of KEGG bacteria in maize rhizosphere soil.

KEGG functional enzyme prediction analysis of microbial gene abundance revealed significant hierarchical differences in the abundance of fungal genes encoding functional enzymes. 3.6.1.3 (ATP diphosphate hydrolase), 3.2.1.3 (glucan 1, 4-alpha-glucosidase), and 2.7.7.7 (DNA-directed RNA polymerase) were high-abundance core enzymes shared by all samples, while enzymes such as 3.4.25.1 (Proteasome endopeptidase complex) and 2.3.1.48 (Histone acetyltransferase) had significantly lower abundance. The expression profile of fungal functional enzymes was considerably altered by the genotype of maize: 2.3.1.48 was particularly concentrated in ZD958; core fungal enzyme abundance was high in WG305, XY335, and QF876; overall enzyme abundance was moderate in JK968, YF303, and DD1104. Fungal metabolic function is significantly influenced by genotype (Figure 5a). The core high-abundance enzymes of rhizosphere bacteria in different maize varieties were mainly 2.7.7.7, 1.6.5.3 (NADH dehydrogenase), and 3.6.4.12 (DNA helicase). Among them, 6.3.5.7 (Glutamine-hydrolysing) had the lowest abundance. The high-abundance bacterial enzymes were evenly distributed among the seven maize varieties, with no obvious variety-specific enrichment; only the low-abundance enzymes, such as 6.3.5.6 and 6.3.5.7, showed weak genotypic differentiation. The abundance of these enzymes was slightly increased in ZD958 and JK968, and the host genotype had a lesser effect on bacterial metabolic function (Figure 5b).

### 3.6. Correlation Between Rhizosphere Microorganisms and Soil Physicochemical Properties of Different Maize Varieties

**Figure 6:**
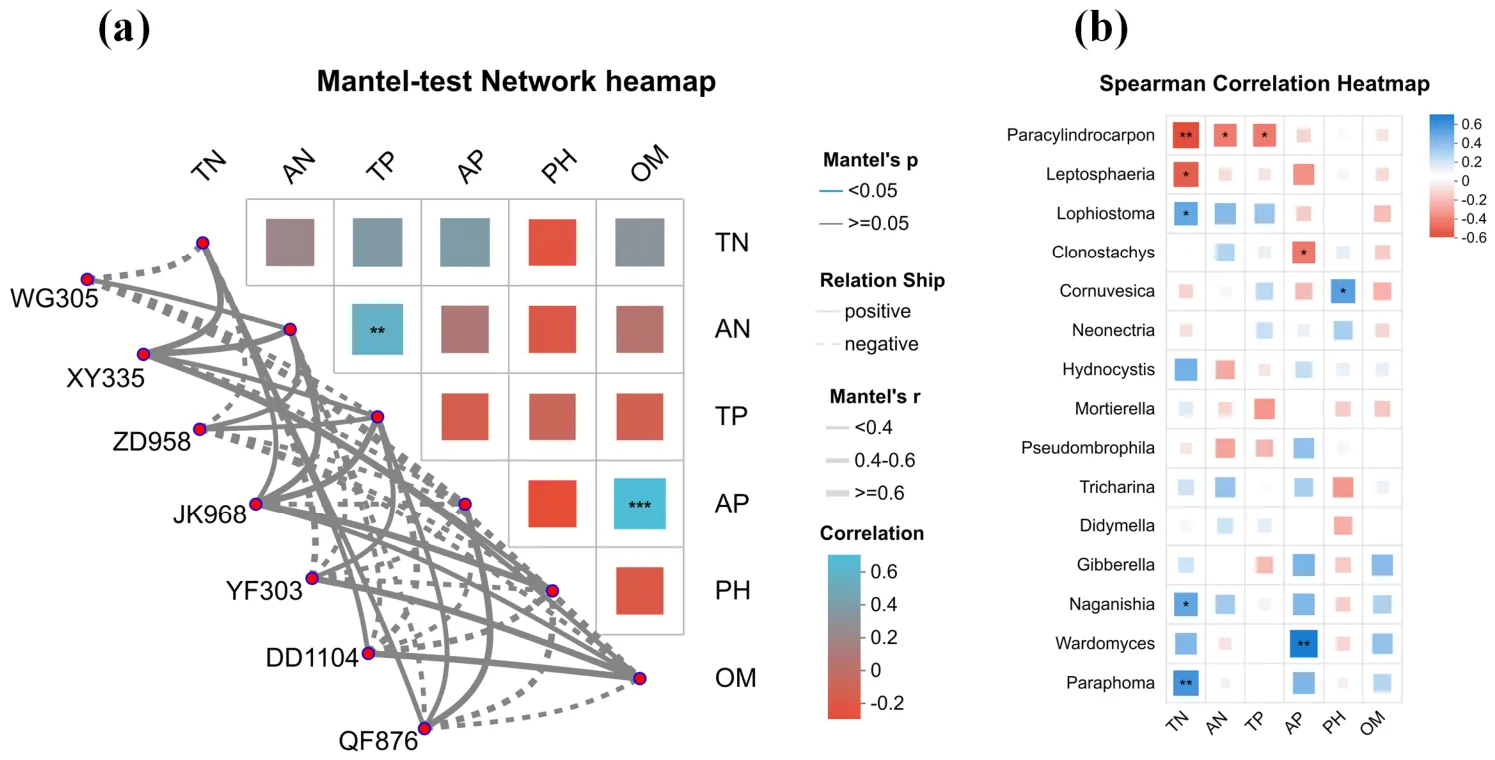
Correlation between rhizosphere soil physicochemical factors and rhizosphere soil fungi in maize (* : 0.01 < *p* ≤ 0.05, ** : 0.001 < *p* ≤ 0.01, *** : *p* ≤ 0.001). (**a**) Mantel-test Network of correlations between rhizosphere soil physicochemical factors and fungal community in maize. (**b**) Heatmap of the correlation between rhizosphere soil physicochemical factors and rhizosphere fungi in maize.

The Mantel test and Spearman correlation analysis revealed that the primary environmental factors controlling the general structure of the fungal community in the maize rhizosphere were available phosphorus (*p* < 0.001) and alkaline-available nitrogen (*p* < 0.01), whereas total nitrogen and organic matter had no discernible impact (Figure 6a). *Paracylindrocarpon* is an indicator group for high nitrogen and phosphorus conditions, and the dominant fungi‌ were generally strongly positively associated with soil nitrogen and phosphorus. Each fungal genus responded differently to nutrient gradients; the abundance of fungal genera was not significantly regulated by pH or organic matter (Figure 6b). This suggests that the genotype of maize and soil nitrogen and phosphorus nutrients interact to induce differentiation in rhizosphere fungal community composition.

**Figure 7:**
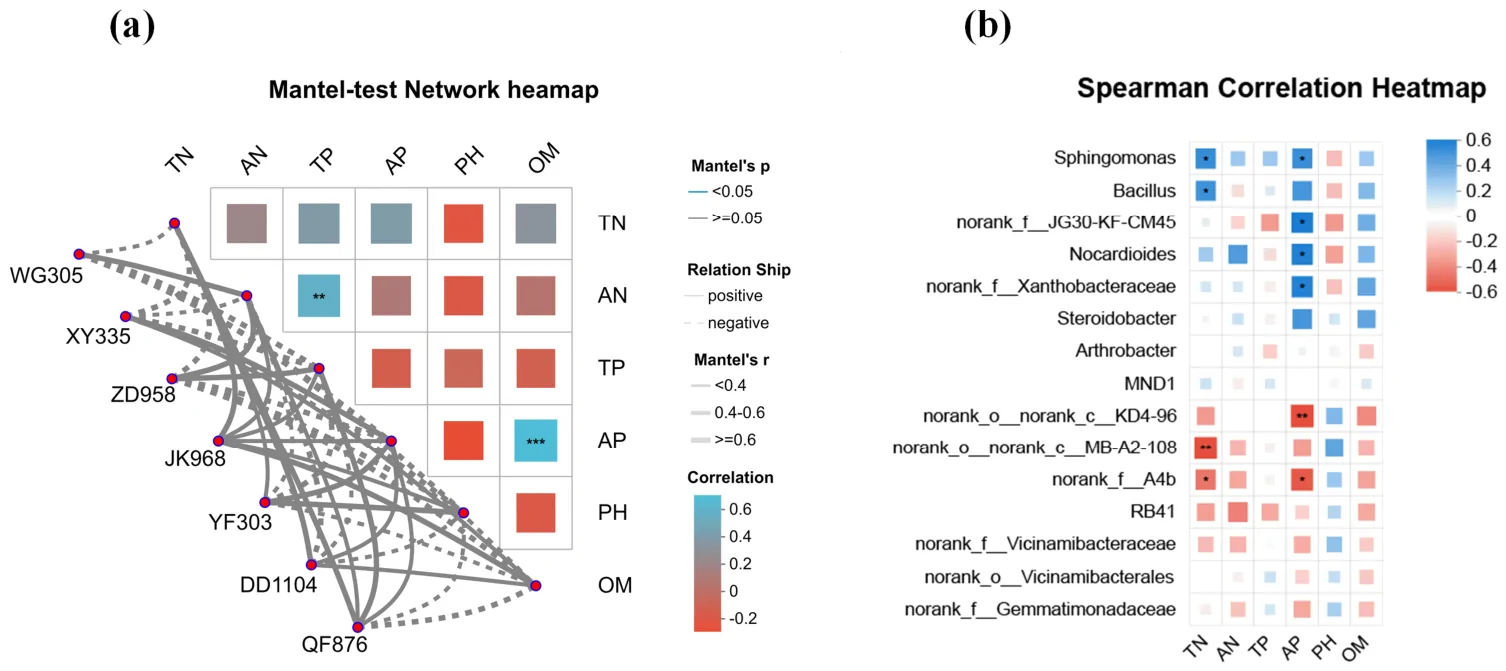
Correlation between rhizosphere soil physicochemical factors and rhizosphere bacteria in maize (* : 0.01 < *p* ≤ 0.05, ** : 0.001 < *p* ≤ 0.01, *** : *p* ≤ 0.001). (**a**) Mantel test network diagram of the correlation between rhizosphere soil physicochemical factors and bacterial community in maize. (**b**) Heatmap of the correlation between rhizosphere soil physicochemical factors and rhizosphere bacteria in maize.

The regulation mechanisms of soil physicochemical variables on bacterial communities in the rhizosphere of maize were examined using the Mantel test and Spearman correlation analysis. The findings demonstrated that total nitrogen and organic matter had no discernible impact on the overall structural differentiation of the bacterial population, although accessible phosphorus (*p* < 0.001) and alkaline-available nitrogen (*p* < 0.01) were important environmental determinants (Figure 7a). Oligotrophic groups RB41 and norank_f_Vicinamibacteraceae displayed a negative association with soil nitrogen and phosphorus nutrients, whereas dominant functional bacterial genera (*Bacillus*, *Sphingomonas*, and *Nocardioides*) exhibited a strong positive correlation (Figure 7b). The abundance of bacteria was only slightly impacted by pH and organic matter. The rhizosphere bacterial community composition was synergistically shaped by soil nitrogen and phosphorus levels and the maize genotype, as evidenced by distinct responses of different bacterial groups to nutrient gradients.

## 4. Discussion

### 4.1. The Connection Between Maize Genotype Response and Soil Physicochemical Properties

According to this study, the rhizosphere soil pH of the seven maize seed types ranged from 7.96 to 8.10, generally showing a slightly alkaline pH, consistent with the fundamental features of irrigated desert soils in Gansu Province’s Hexi Corridor [43,44]. While total nitrogen (TN), available nitrogen (AN), and available phosphorus (AP) demonstrated considerable varietal differences, total phosphorus (TP) and soil organic matter did not differ significantly between cultivars. The rhizosphere’s TN concentration was highest in WG305 and DD1104, AN was much greater in ZD958 and DD1104 than in XY335 and QF876, and AP was significantly higher in YF303 and WG305 (Table 1). Previous studies have confirmed that different maize genotypes can significantly alter rhizosphere nutrient availability by regulating the types of carbon sources in root exudates (e.g., the secretion ratios of organic acids, sugars, and amino acids) and the amount of root abscission [45]. In this study, XY335 had the lowest available nitrogen and available phosphorus, combined with its lowest mean organic matter content, suggesting that this variety may have lower root carbon input or weaker rhizosphere nutrient-activation capacity. In contrast, WG305 showed outstanding performance in both total nitrogen and available phosphorus, possibly related to its stronger root exudation strategy or nitrogen assimilation capacity. Recent studies on the response of rhizosphere microbial communities of two maize hybrids to different nitrogen application levels have also shown that nitrogen availability is a key factor driving changes in rhizosphere microbial community structure, consistent with this study’s results [46]. It is noteworthy that the significant differences in available phosphorus and alkaline nitrogen among varieties not only reflect the plants’ active regulation of nutrients, but also, as found in studies of wheat-maize rotation systems, soil nutrient availability is a decisive factor driving the variation and functional differentiation of rhizosphere microbial communities in wheat and maize. The significant differences in available phosphorus and alkaline nitrogen among the varieties in this study (*p* < 0.05) provide an important environmental background for the subsequent analysis of microbial community structure [47,48,49].

### 4.2. Differential Regulation of Rhizosphere Microbial Alpha Diversity by Maize Varieties

At the phylum level, there were no significant differences in species richness indices (Sobs and Bootstrap) of the rhizosphere fungal communities among the seven varieties. However, significant varietal differentiation was observed in the Shannon and Simpson indices, indicating that maize varieties have a much greater impact on the evenness and diversity of the fungal community than on the total number of taxa (Table 2). This result suggests that different maize varieties may exert selective pressure on the rhizosphere fungal community by releasing different signaling molecules or antimicrobial compounds, thereby altering the relative abundance distribution within the community [50]. At the genus level, fungal species richness varied considerably among the varieties. JK968 and DD1104 showed significantly higher Sobs and Bootstrap indices than the other five varieties. Their Shannon indices were also significantly higher, whereas their Simpson indices were significantly lower (Table 3). These findings suggest that JK968 and DD1104 support richer, more diverse, and more homogeneous fungal community structures. Such a community structure may enhance the functional redundancy and disturbance resistance stability of the rhizosphere microecosystem. Previous studies have indicated that plant genotypes can selectively enrich or suppress specific fungal communities by regulating the chemical composition and quantity of root exudates [51,52,53]. The high abundance and diversity of rhizosphere fungal communities observed in JK968 and DD1108 in this study may be attributed to a greater capacity to supply carbon sources or to more robust rhizosphere interaction signaling networks.

The bacterial and fungal communities exhibited quite different response patterns. At the phylum level, XY335 had the lowest Simpson index (i.e., ideal community evenness) and the highest Shannon diversity index, whereas ZD958 had the highest species richness index. JK968 had the highest Shannon diversity index, while DD1104 had the best species richness at the genus level (Table 4). This asynchronous response pattern in bacterial and fungal diversity across various kinds may be due to variations in community assembly mechanisms and sensitivity to environmental cues. Research on how domestication and breeding of maize affect rhizosphere microbial recruitment has shown that contemporary maize hybrids exhibit distinct rhizosphere microbial recruitment strategies compared with their primordial predecessors [54,55].

### 4.3. Core Rhizosphere Microbiota and Variety Specificity in Maize Varieties

The Venn diagram analysis in this study showed that the proportions of shared taxa in the rhizosphere fungal and bacterial communities of maize were very high, at 50% and 82.50%, respectively, with only a few varieties containing a single endemic phylum. In contrast, at the genus level, the cross-variety specificity of the microbial community was more pronounced: fungal genus specificity was 14.13%, with each maize variety containing 8–26 endemic fungal genera; bacterial genus specificity was 47.38%, with each maize variety containing 11–23 endemic bacterial genera (Figure 1). These results indicate that maize rhizosphere fungal communities exhibit high conservation and stability, with their core components less affected by genotypic disturbances, whereas bacterial communities exhibit high varietal plasticity, reflecting their sensitive response to changes in host root exudate composition. Studies have long confirmed that plant genotypes can strongly modify the structure and growth of maize rhizosphere microbial communities [56,57]. This study further reveals significant differences in community conservation between bacteria and fungi by using a larger number of varieties (7 mainstream seed production varieties) and higher taxonomic resolution (phylum and genus levels).

Ascomycota was the most prevalent fungal phylum in the rhizosphere of all maize varieties in this study (relative abundance > 60%), which is consistent with research on the organization of fungal communities in grassland and farmland environments worldwide [58]. The second most prevalent groups were Basidiomycota and Mortierellomycota. Members of Mortierellomycota are crucial for soil carbon cycling and lipid metabolism [59], and variations in their abundance may be directly linked to the transformation of soil organic matter. Proteobacteria are mostly r-strategy groups that rapidly proliferate in response to root exudates [60]; *Acidobacteria* are biased towards K-strategy, with a competitive advantage under oligotrophic conditions [61]; and Chlorobacteria play an important role in organic matter decomposition and carbon cycling [62]. The pH of all maize rhizosphere soils in this study ranged from 7.96 to 8.10, indicating a slightly alkaline environment. However, *Acidobacteriota* was one of the three dominant bacterial phyla (Figure 2b). This contradicts the traditional understanding that *Acidobacteriota* thrive in acidic environments with a pH of 3.0–6.5. This may be due to significant physiological differentiation among the *Acidobacteriota* group, as well as the targeted screening effect of long-term drip irrigation fertilization [63].

The common dominant fungal genera at the genus level are *Leptosphaeria*, *Mortierella*, and *Paracylindrocarpon*. Compared to other varieties, ZD958 shows a substantially higher overall abundance of the main genera *Paracylindrocarpon* and *Leptosphaeria*. The northern corn leaf blight pathogen, *Leptosphaeria*, is prevalent in ZD958’s rhizosphere, warranting particular attention because it may indicate this variety is at risk of diseases [64]. In contrast, JK968 has a more evenly distributed, typically lower-abundance fungal community, indicating a more balanced rhizosphere fungal community structure and possibly greater disease resistance.

At the bacterial genus level, the top 15 genera include a large number of norank taxa and typical rhizosphere functional fungal genera, with norank_f_Vicinamibacteraceae, *Arthrobacter*, RB41, and *Bacillus* being the core dominant taxa. In DD1104, the relative abundance of *Arthrobacter* is much higher than in other varieties (Figure 3); *Arthrobacter* has been reported to have growth-promoting and biocontrol functions [65,66]. XY335 showed the highest total abundance of dominant bacterial genera, with significant enrichment of *Bacillus*. *Bacillus* was detected in the rhizosphere of all varieties, consistent with its widespread distribution as a classic PGPR bacterium in the maize rhizosphere. Recent studies have shown that changes in the abundance of growth-promoting bacteria, such as *Bacillus*, are closely associated with the nitrogen and phosphorus uptake efficiency of maize varieties [67,68], providing a new perspective on the functional explanation of bacterial community differences in this study.

### 4.4. Differential Microorganisms in the Rhizosphere Soil of Different Maize Varieties

The distinct differential microbial marker communities of each maize variety were further identified in this study using LEfSe analysis (LDA > 3.5), with WG305 showing enrichment for Wardomyces and Paramyrothecium; XY335 was enriched in *Monascus* and *Paracylindrocarpon*; ZD958 was enriched in *Sordariomycetes*, Pleosporales, and Dothideomycetes, with Sordariomycetes exhibiting the strongest indicative effect and highest LDA score; JK968 was enriched in Basidiomycota and Agaricomycetes; YF303 was enriched in Botrytis; and QF876 was enriched in Gibberella. The great selectivity of maize genotypes for rhizosphere fungal communities was further confirmed by the distinct indicator fungus that each variety displayed across taxonomic ranks (Figure 4a). Bacterial LEfSe analysis also revealed clear variety specificity: WG305 was characterized by key indicator bacteria from the phylum Chlorella, order Thermomicrophytes, and Nitrolancea; XY335 was enriched in Gemmatimonadota and Methylomirabilota; ZD958 was enriched in the class Anaerolineae and several unclassified groups; JK968 was specifically enriched in Micromonosporaceae and Micromonospora; YF303 was enriched in thermophilic actinomycetes; DD1104 used *Sphingomonas* as a marker group; and QF876 used Sphingomonasceae as a marker differential group (Figure 4b). The discovery of these indicator groups provides important biomarkers for the precise regulation of variety-microbe interactions. For example, *Micromonospora* promotes plant growth and improves nitrogen use efficiency [69], and its enrichment in JK968’s rhizosphere may partially explain this variety’s excellent rhizosphere microbial diversity. *Sphingomonas* has been reported to have dual functions: degrading organic matter and promoting plant stress resistance [70,71]; its enrichment in DD1104 may confer specific environmental adaptability to this variety. While the highly abundant *Anaerolineae* in ZD958 harbor potential plant pathogens, they also include a large number of *Clonostachys* with biocontrol activity, which have been extensively studied as biocontrol agents [72,73]. Divergence in the rhizosphere microbial community is mediated by a combination of regional irrigation patterns, soil spatial variability, and maize root shape [74,75]. Natural soil heterogeneity in the Hexi Corridor desert irrigation area is influenced by the uneven distribution of nutrients and soil particles [43]. The competitive advantage of oligotrophic bacteria such as Acidobacterium and Mortierellomycota was altered by alternating drought and drip irrigation, which increased variation in rhizosphere oxygen partial pressure [76]. The carbon source supply of rhizosphere microorganisms is directly affected by the acids, soluble sugars, and amino acids secreted by the roots, which can selectively filter out bacteria and fungi [77]. This study found that DD1104 and JK968 have well-developed fibrous roots and higher rhizosphere carbon input, which are closely coupled with their genus-level fungal richness and beneficial actinomycete enrichment. In contrast, XY335 has a weaker nutrient-activation capacity from root exudates, significantly lower rhizosphere-available nitrogen and phosphorus, and limited microbial community diversity.

### 4.5. KEGG Functional Profile of Maize Rhizosphere Microorganisms

KEGG enzyme prediction of fungal gene abundance in maize rhizosphere soil showed that EC numbers 3.6.1.3 (ATP diphosphate hydrolase), 3.2.1.3 (glucan 1, 4-alpha-glucosidase), and 2.7.7.6 (DNA-directed RNA polymerase) were high-abundance core enzymes shared throughout the entire sample, reflecting the maintenance of basic metabolic functions in the fungal community. While JK968, YF303, and DD1104 had moderate overall enzyme abundance, WG305, XY335, and QF876 retained high levels of core fungal enzyme abundance. This suggests that fungal metabolic processes are significantly regulated by different types of maize. The high-abundance enzymes in the bacterial core were mainly 2.7.7.7 (DNA-directed RNA polymerase), 1.6.5.3 (NADH dehydrogenase), and 3.6.4.12 (DNA helicase). In contrast, enzymes such as 6.3.5.6 and 6.3.5.7 (Glutamine-hydrolysing) had the lowest abundance. High-abundance bacterial enzymes showed highly uniform distribution across the seven maize varieties, with no significant variety-specific enrichment. Only low-abundance enzymes, such as 6.3.5.6 and 6.3.5.7, showed weak genotypic differentiation, while their abundance increased slightly in ZD958 and JK968. This indicates that, compared with fungal communities, the metabolic functions of bacterial communities are less affected by maize genotype perturbations. This may be because the core metabolic functions of bacterial communities are mainly driven by genes encoding higher-abundance core enzymes, and the conservation of these core functions ensures the high stability of bacterial metabolism across different rhizosphere varieties. Variety-specific variation in low-abundance enzymes may reflect the fine-grained regulatory effects of the rhizosphere microenvironment on bacterial metabolic pathways in each variety. This finding is consistent with previous findings regarding the high functional redundancy of bacterial metabolic functions [78].

### 4.6. Interactions Between Soil Physicochemical Properties and Microbial Communities

Mantel tests and Spearman correlation analyses are powerful tools for detecting relationships between microbial communities and environmental factors. Our results show that available phosphorus (*p* < 0.001) and available nitrogen (*p* < 0.01) are the core environmental factors regulating the overall structure of the maize rhizosphere fungal community. Dominant fungal genera (such as *Paracylindrocarpon* and *Mortierella*) are generally significantly positively correlated with soil nitrogen and phosphorus nutrients [79,80]. This indicates that soil nitrogen and phosphorus nutrient levels, along with maize genotypes, synergistically drive the compositional differentiation of the rhizosphere fungal community. Notably, pH and organic matter have a weak regulatory effect on fungal genus abundance. This result may be related to the experimental background of the overall weakly alkaline soil and insignificant differences in organic matter in the study field. Under these conditions, changes in the availability of phosphorus and nitrogen become the primary ecological drivers.

For the bacterial community, Mantel tests and Spearman analyses also show that available phosphorus (*p* < 0.001) and available nitrogen (*p* < 0.01) are key environmental variables driving community differentiation. Dominant functional bacterial genera (*Bacillus*, *Sphingomonas*, *Nocardioides*) showed a significant positive correlation with nitrogen and phosphorus nutrient availability, while oligotrophic groups (RB41, norankf. Vicinamibacteraceae) showed a negative correlation with nutrient gradients. This “nutrient preference” pattern reveals the dynamic balance among different ecological strategy groups in the rhizosphere microdomain. These findings are highly consistent with conclusions that nitrogen availability drives the assembly of the maize rhizosphere microbial community and echo recent research showing that plant genotypes shape the rhizosphere microbial community by regulating nitrogen and phosphorus availability [46,81]. This study provides an important theoretical framework for understanding the interactions between seed maize varieties and the rhizosphere microecology.

## 5. Conclusions

This study used rhizosphere soils from seven major maize varieties at the Linze Seed Production Base in the Hexi Corridor as the research object to systematically analyze the regulatory mechanisms of variety genotypes on rhizosphere soil physicochemical properties, microbial communities, and metabolic functions. The overall rhizosphere soils of the tested maize were weakly alkaline. Significant differences were observed among varieties in total nitrogen, available nitrogen, and available phosphorus, while organic matter and total phosphorus were only slightly affected by variety. Maize genotypes significantly altered the alpha diversity of rhizosphere fungi and bacteria. DD1104 and JK968 showed the highest rhizosphere fungal community richness and overall diversity, ZD958 had the highest species abundance at the phylum level, and DD1104 exhibited the highest species richness at the genus level. The fungal community showed a high proportion of shared taxa across varieties, indicating strong community conservation. There were many bacterial taxa, and the consequences of genotype-driven differentiation were more pronounced. For fungi and bacteria, respectively, the core dominating phyla were Ascomycota and Proteobacteria. Different rhizosphere microbiota were identified through host-genotype-directed screening, and LEfSe analysis confirmed that each maize variety was enriched with specific indicator bacteria. Fungal metabolic function is significantly regulated by variety, while bacterial core metabolic function tends to stabilize. Available phosphorus and alkaline-available nitrogen are key nutrient factors driving the differentiation of rhizosphere fungal and bacterial communities. Nitrogen and phosphorus nutrients, along with maize genotype, synergistically shape the rhizosphere microbial community structure. In conclusion, this study provides a theoretical foundation for selecting high-quality seed maize varieties and for controlling rhizosphere soil microecology in the Hexi Corridor by regulating soil nitrogen and phosphorus levels and selectively targeting rhizosphere-specific microorganisms through genotype regulation of seed maize.

## Figures and Tables

**Table 1:** Physicochemical factor indicators of rhizosphere soil of different maize varieties.

Sample	TN	AN	TP	AP	pH	OM
	(%)	(mg/Kg)	(%)	(mg/Kg)		(g/Kg)
WG305	0.04 ± 0.00 a	68.17 ± 20.32 abc	0.06 ± 0.00 a	20.31 ± 4.14 a	8.02 ± 0.10 a	17.93 ± 4.29 a
XY335	0.02 ± 0.00 c	45.90 ± 19.76 c	0.04 ± 0.02 a	4.75 ± 0.88 c	8.03 ± 0.16 a	13.92 ± 1.20 a
ZD958	0.02 ± 0.01 c	84.70 ± 21.70 a	0.08 ± 0.02 a	12.97 ± 3.31 b	8.10 ± 0.06 a	14.98 ± 3.14 a
JK968	0.03 ± 0.01 b	81.45 ± 3.71 ab	0.06 ± 0.01 a	14.17 ± 2.35 b	8.02 ± 0.03 a	16.15 ± 1.39 a
YF303	0.02 ± 0.00 c	71.97 ± 6.46 abc	0.05 ± 0.01 a	20.94 ± 1.99 a	7.96 ± 0.06 a	17.18 ± 1.05 a
DD1104	0.04 ± 0.00 a	84.57 ± 6.09 a	0.07 ± 0.00 a	16.39 ± 1.58 ab	8.05 ± 0.06 a	14.77 ± 1.40 a
QF876	0.02 ± 0.00 bc	55.24 ± 8.76 bc	0.20 ± 0.26 a	17.50 ± 2.10 ab	8.09 ± 0.03 a	15.83 ± 1.46 a

The mean ± standard error (SE) (n = 3; Duncan’s multiple range test) is used to present all data. At *p* < 0.05, means in the same column with the same letter do not differ substantially.

**Table 2:** Alpha diversity in rhizosphere soil fungi of different maize varieties at the phylum level.

Sample	Sobs	Shannon	Simpson	Bootstrap
WG305	9.33 ± 0.58 a	0.70 ± 0.15 bc	0.71 ± 0.07 a	9.33 ± 0.58 a
XY335	9.00 ± 1.00 a	0.82 ± 0.12 ab	0.57 ± 0.11 b	9.12 ± 0.87 a
ZD958	8.00 ± 1.73 a	0.56 ± 0.06 c	0.75 ± 0.04 a	8.12 ± 1.64 a
JK968	9.33 ± 0.58 a	0.86 ± 0.12 ab	0.57 ± 0.08 b	9.38 ± 0.66 a
YF303	9.67 ± 0.58 a	0.72 ± 0.08 abc	0.66 ± 0.05 ab	9.71 ± 0.62 a
DD1104	9.33 ± 0.58 a	0.93 ± 0.07 a	0.54 ± 0.05 b	9.35 ± 0.61 a
QF876	8.00 ± 1.00 a	0.72 ± 0.13 abc	0.65 ± 0.07 ab	8.26 ± 1.38 a

The mean ± standard error (SE) (n = 3; Duncan’s multiple range test) is used to present all data. At *p* < 0.05, means in the same column with the same letter do not differ substantially.

**Table 3:** Alpha diversity in rhizosphere soil fungi of different maize varieties at the genus level.

Sample	Sobs	Shannon	Simpson	Bootstrap
WG305	94.67 ± 6.35 c	3.26 ± 0.03 a	0.08 ± 0.02 ab	95.45 ± 6.74 c
XY335	100.67 ± 16.74 c	2.60 ± 0.65 b	0.20 ± 0.16 a	103.09 ± 19.29 c
ZD958	94.67 ± 2.52 c	2.63 ± 0.33 b	0.15 ± 0.07 ab	96.48 ± 3.11 c
JK968	153.67 ± 10.60 a	3.45 ± 0.15 a	0.06 ± 0.02 b	157.27 ± 12.99 a
YF303	132.33 ± 6.51 b	3.57 ± 0.16 a	0.05 ± 0.02 b	134.41 ± 7.10 b
DD1104	153.00 ± 3.61 a	3.57 ± 0.11 a	0.05 ± 0.01 b	155.50 ± 4.06 a
QF876	126.33 ± 12.06 b	3.37 ± 0.22 a	0.07 ± 0.03 ab	128.69 ± 13.50 b

The mean ± standard error (SE) (n = 3; Duncan’s multiple range test) is used to present all data. At *p* < 0.05, means in the same column with the same letter do not differ substantially.

**Table 4:** Alpha diversity in rhizosphere soil bacteria of different maize varieties at the phylum level.

Sample	Sobs	Shannon	Simpson	Bootstrap
WG305	34.67 ± 0.58 b	2.15 ± 0.04 bc	0.17 ± 0.01 bcd	35.30 ± 0.63 b
XY335	35.00 ± 1.00 ab	2.29 ± 0.05 a	0.14 ± 0.01 d	35.57 ± 0.96 ab
ZD958	36.67 ± 1.15 a	2.21 ± 0.04 ab	0.16 ± 0.01 cd	37.47 ± 1.13 a
JK968	34.67 ± 0.58 b	2.02 ± 0.04 de	0.19 ± 0.01 ab	34.92 ± 0.81 b
YF303	34.33 ± 1.53 b	1.94 ± 0.08 e	0.21 ± 0.03 a	34.65 ± 1.55 b
DD1104	35.33 ± 1.15 ab	2.07 ± 0.02 cd	0.19 ± 0.01 abc	35.67 ± 1.43 ab
QF876	35.67 ± 0.58 ab	2.03 ± 0.05 de	0.19 ± 0.02 ab	35.91 ± 0.62 ab

The mean ± standard error (SE) (n = 3; Duncan’s multiple range test) is used to present all data. At *p* < 0.05, means in the same column with the same letter do not differ substantially.

**Table 5:** Alpha diversity in rhizosphere soil bacteria of different maize varieties at the genus phylum level.

Sample	Sobs	Shannon	Simpson	Bootstrap
WG305	618.33 ± 14.57 cd	4.90 ± 0.24 bcd	0.02 ± 0.01 a	656.66 ± 19.25 bc
XY335	583.33 ± 12.50 e	4.75 ± 0.07 d	0.02 ± 0.00 a	620.67 ± 11.94 d
ZD958	602.00 ± 22.54 de	4.83 ± 0.17 cd	0.02 ± 0.01 a	641.63 ± 28.53 cd
JK968	653.33 ± 14.64 b	5.16 ± 0.10 a	0.01 ± 0.00 a	690.33 ± 16.39 b
YF303	640.67 ± 19.50 bc	5.00 ± 0.07 abc	0.01 ± 0.00 a	682.10 ± 21.95 b
DD1104	690.00 ± 10.82 a	5.02 ± 0.06 abc	0.02 ± 0.00 a	730.93 ± 8.41 a
QF876	634.00 ± 9.54 bc	5.11 ± 0.09 ab	0.01 ± 0.00 a	672.86 ± 13.75 bc

The mean ± standard error (SE) (n = 3; Duncan’s multiple range test) is used to present all data. At *p* < 0.05, means in the same column with the same letter do not differ substantially.

## Data Availability

The original data presented in the study are openly available in the NCBI database with the study accession number PRJNA1493312, that are publicly accessible at https://www.ncbi.nlm.nih.gov/sra, accessed on 11 July 2026.

## Supplementary Materials

The following supporting information can be downloaded at: https://www.mdpi.com/article/10.3390/microorganisms14081746/s1, Table S1: Geographical location of maize rhizosphere soil samples.
