# Supplementary material for: Analysis of Rhizosphere Microbial Diversity Among Different Maize Varieties in the Hexi Corridor
Source: Microorganisms. 2026 Aug 8;14(8):1746. doi: 10.3390/microorganisms14081746 (PMC13515286; doi:10.3390/microorganisms14081746)
Supplement: Supplementary file 1 [file microorganisms-14-01746-s001.zip › microorganisms-4454567-supplementary.pdf]

Supplementary Material

Table S1 Geographical location of maize rhizosphere soil samples

**samples**	**East longitude**	**North latitude**
Wugu 305 (WG305)	100° 26′ 15′′	38° 57′ 4′′
Xianyu 335 (XY335)	100° 26′ 25′′	39° 0′ 7′′
Zhengdan 958 (ZD958)	100° 26′ 22′′	39° 2′ 8′′
Jingke 968 (JK968)	100° 25′ 54′′	38° 54′ 18′′
Yufeng 303 (YF303)	100° 25′30′′	38° 52′ 51′′
Dedan 1104 (DD1104)	100° 23′35′′	38° 53′ 15′′
Qinfeng 87 6(QF876)	100° 22′43′′	38° 55′50′′
